# Augmentation of Glucotoxicity, Oxidative Stress, Apoptosis and Mitochondrial Dysfunction in HepG2 Cells by Palmitic Acid

**DOI:** 10.3390/nu11091979

**Published:** 2019-08-22

**Authors:** Arwa Alnahdi, Annie John, Haider Raza

**Affiliations:** Department of Biochemistry, College of Medicine and Health Sciences, UAE University, P.O. Box-17666, Al Ain, UAE

**Keywords:** glucolipotoxicity, palmitic acid, HepG2 cells, mitochondrial dysfunction, redox metabolism, apoptosis

## Abstract

Hyperglycemia and hyperlipidemia are the hallmarks of diabetes and obesity. Experimental and epidemiological studies have suggested that dietary management and caloric restriction are beneficial in reducing the complications of diabesity. Studies have suggested that increased availability of energy metabolites like glucose and saturated fatty acids induces metabolic, oxidative, and mitochondrial stress, accompanied by inflammation that may lead to chronic complications in diabetes. In the present study, we used human hepatoma HepG2 cells to investigate the effects of high glucose (25 mM) and high palmitic acid (up to 0.3 mM) on metabolic-, inflammatory-, and redox-stress-associated alterations in these cells. Our results showed increased lipid, protein, and DNA damage, leading to caspase-dependent apoptosis and mitochondrial dysfunction. Glucolipotoxicity increased ROS production and redox stress appeared to alter mitochondrial membrane potential and bioenergetics. Our results also demonstrate the enhanced ability of cytochrome P450s-dependent drug metabolism and antioxidant adaptation in HepG2 cells treated with palmitic acid, which was further augmented with high glucose. Altered NF-kB/AMPK/mTOR-dependent cell signaling and inflammatory (IL6/TNF-α) responses were also observed. Our results suggest that the presence of high-energy metabolites enhances apoptosis while suppressing autophagy by inducing inflammatory and oxidative stress responses that may be responsible for alterations in cell signaling and metabolism.

## 1. Introduction

Most of the complications associated with metabolic disorders, including type 2 diabetes, obesity, insulin resistance, non-alcoholic fatty liver diseases (NAFLD), and non-alcoholic associated steatohepatitis (NASH) are associated with hyperglycemia and increased free saturated fatty acid concentration in the blood [1,2]. Glucotoxicity and lipotoxicity due to excess caloric intake and increased nutrient availability are the hallmarks of diabetes progress and disease complications [3,4,5,6]. However, under in vivo conditions, due to a series of physiological and metabolic communications associated with different tissues under the influence of dietary and endocrine regulations, it is difficult to distinguish between the effects of glucotoxicity and lipotoxicity. Saturated and unsaturated fatty acids are known to have differential effects on cell death and survival, though the mechanisms associated with these differences are unknown. Palmitic acid (PA) is the most abundant type of saturated fatty acid in the plasma, and has been implicated in the toxicity of pancreatic β-cells, hepatocytes, and many other cells [7,8,9,10,11]. PA has also been reported to enhance cellular oxidative stress and apoptosis by suppressing cytoprotective autophagy. In contrast, unsaturated fatty acids, such as oleic acid, promote autophagy but have minimal effects on apoptosis [12,13,14,15]. Electron microscopy (EM )studies have indicated that pancreatic islet cells treated with high glucose/PA showed increased accumulation of autophagosomes, vacuolar changes, damaged mitochondria, and endoplasmic reticulum distention, accompanied by an increased expression of mTORC1, an inhibitor of autophagy, causing increased metabolic stress and apoptosis [16,17]. Similarly, nutrient overload with high glucose for prolonged periods increases mitochondrial reactive oxygen species (ROS) generation in cells, leading to severe perturbation of metabolism and cell signaling. Increased glucolipotoxicity has been implicated with mitochondrial dysfunction and endoplasmic reticulum stress and inflammation in diabetes- and obesity-associated complications [18,19]. The underlying mechanism, however, is still not clear. Functional deficiency associated with energy metabolism and oxidative stress is the main cause of abnormal insulin secretion and action. A novel approach to treating these abnormalities is highly relevant in nutrient-regulated diabetes/obesity management. Increased fatty acids inhibit insulin signaling by altering Akt and protein tyrosine phosphatase 1B-dependent pathways, which is considered a potential mechanism for insulin resistance and cardiovascular complications in diabetes [20,21]. High glucose (HG) and high fatty acid (HFAs) are the characteristics of diabetic complications associated with increased oxidative stress and inflammation-linked endothelial and mitochondrial dysfunction [22,23,24]. AMPK is a serine/threonine kinase that stimulates catabolic processes and inhibits anabolic processes when cellular energy levels are low. However, it has been shown that under nutrient-rich conditions, AMPK activity is diminished and becomes uncoupled from autophagy, leading to increased apoptosis [22].

Therapeutic intervention to target the autophagy/apoptosis machinery that ultimately reprograms the cellular metabolism under nutrient overload conditions may therefore provide strategies for better management and treatment of diabesity-associated complications. Augmentation of PA-induced-lipotoxicity by HG, or vice versa, is controversial, as actual in vivo concentrations of glucose and PA under conditions of diabetes, fasting or after meal, in normal and obese subjects have not yet been defined conclusively, and might be highly variable due to various physiological and endocrine factors involved in regulating the bioavailability of nutrients. Therefore, we designed in vitro experiments to determine the molecular mechanisms of action of glucose and PA overload in HepG2 cells. Our aim was to elucidate the mechanism of glucotoxicity alone (25 mM) or in combination with high PA (up to 0.3 mM) under in vitro conditions. We have shown that mitochondrial respiratory function, energy/drug metabolism, redox homeostasis, and cellular oxidative stress are affected in glucolipotoxicity. These changes appear to be associated, at least in part, with altered inflammatory and NF-kB/mTOR/AMPK-dependent cell signaling pathways. Furthermore, in the presence of HG, PA treatment augments glucotoxicity, as shown by the reduction in autophagy with increased apoptosis in HepG2 cells.

## 2. Materials and Methods

### 2.1. Materials

Palmitic acid, fatty acid-free bovine serum albumin (BSA), 3-(4,5-dimethylthiazol-2-yl)-2,5-diphenyltetrazolium bromide (MTT), reduced and oxidized glutathione, 1-chloro 2,4-dinitrobenzene (CDNB), cumene hydroperoxide, glutathione reductase, glucose-6-phosphate, NADH, NADPH, cytochrome c, coenzyme Q2, antimycin A, dodecyl maltoside, N-nitrosodimethylamine(NDMA), erythromycin,7-ethoxyresorufin, methoxyresorufin, resorufin, Hoechst 33342, Oil Red O stain, and kits for ATP and hexokinase (HK )were purchased from Sigma (St Louis, MO, USA). 2′, 7′-Dichlorofluorescein diacetate (DCFDA) was procured from Molecular Probes (Eugene, OR, USA). Kits for nitric oxide and mitochondrial membrane potential assays were purchased from R & D Systems (Minneapolis, MN, USA), while those for lipid peroxidation (LPO) and aconitase were procured from Oxis Int, Inc. (Portland, OR, USA). Kits for GSH/GSSG assays were procured from Promega Corp. (Madison, WI, USA). Apoptosis detection kits for flow cytometry and IL6 and TNF-α measurement kits were purchased from BD Pharmingen (BD Biosciences, San Jose, CA, USA). Kits for catalase and protein carbonylation assays were purchased from Cayman Chemical (Ann Arbor, MI, USA). Kits for superoxide dismutase (SOD) were purchased from Trevigen (Gaithersburg, MD, USA), while those for glutamate dehydrogenase (GDH were purchased from Abcam (Cambridge, UK). HepG2 cells were purchased from American Type Culture Collection (Manassas, VA, USA). Polyclonal antibodies against PARP, caspase-3, NFκB, and beta-actin were procured from Santa Cruz Biotechnology Inc. (Santa Cruz, CA, USA) while those for mTOR, p-mTOR, AMPK, and p-AMPK were purchased from Cell Signaling Technology, Inc. (Danvers, MA, USA). Polyclonal antibodies against CYP2E1 and CYP3A4 were purchased from Aviva Systems Biology (San Diego, CA, USA). Reagents for cell culture, SDS-PAGE, and western blot analysis were purchased from Gibco BRL (Grand Island, NY, USA) and Bio-Rad Laboratories (Richmond, CA, USA).

### 2.2. Cell Culture, Treatment and Fractionation

HepG2 cells were cultured in DMEM supplemented with 2 mM glutamine, 10% fetal calf serum, and non-essential amino acids in the presence of 5% CO_2_ at 37 °C. Cells were cultured to 80% confluence and then treated with normal (5.5 mM) (which is close to the in vivo fasting value) or high glucose (25 mM) (to mimic the in vivo diabetic condition) with/without high fatty acids (up to 0.3 mM palmitic acid) for 24 h. A 100 mM stock solution of palmitic acid was prepared in warm ethanol and then conjugated to 1% fatty acid free-BSA in a molar ratio of 6:1. The cells were treated with different concentrations of palmitate by adding appropriate amounts of the palmitate/BSA conjugate to the cultured cells in DMEM media supplemented with 1% FBS. Control cells for normal and high glucose media were treated with vehicle (BSA/ethanol) alone. Concentrations and time points used for high glucose or high fatty acid treatments were based on the MTT-based cytotoxicity studies and literature search. After the desired time of treatment, cells were harvested, washed with PBS (pH 7.4) and homogenized in H-medium buffer (70 mM sucrose, 220 mM mannitol, 2.5 mM HEPES, 2 mM EDTA, and 0.1 mM phenylmethylsulfonyl fluoride, pH 7.4) at 4 °C. Cellular fractionation to prepare mitochondria and postmitochondrial (PMS) fractions was performed by centrifugation, as described previously [25,26,27,28].

### 2.3. Measurement of Cell Survival

MTT assay was used to determine the glucolipotoxicity induced in the presence of HG/HFA, using the mitochondrial enzyme based cellular viability test as described in our previous study [27,28].

### 2.4. Measurement of ROS, LPO, Protein Peroxidation, DNA Damage, and Apoptosis

Intracellular production of ROS was measured flurometrically using the cell permeable probe, DCFDA, which preferentially measures peroxides. Briefly, treated and control cells (~1 × 10^5^ cells/mL) were grown on cover slips and incubated with 5 µM DCFDA for 30 min at 37 °C. Cells were washed twice with PBS, and the fluorescence analyzed microscopically, as described above [28]. DCFDA-based ROS assay was also performed fluorimetrically using the same probe and measured using the ELISA reader (TECAN Infinite M 200 PRO, Austria) at an excitation wavelength of 488 nm and an emission wavelength of 525, nm and also by FACS analysis, as described previously [25,26,27].

Lipid peroxidation (LPO) in the cell lysate from HG/HFA-treated and control cells was measured using the LPO-586 kit as per the manufacturer’s protocol and the concentration of MDA calculated from the standard curve as described previously [27,28].

Protein carbonylation, an oxidative modification of proteins under oxidative stress conditions was measured by dinitrophenylhydrazine (DNPH) coupling method according to the vendor’s protocol as described previously [29]. Briefly, total lysates from HG/HFA-treated and control cells were treated with DNPH dissolved in 2N HCl for one hour. The TCA-precipitated proteins were washed with ethanol–ethyl acetate (1:1), and the DNPH-coupled proteins were then measured spectrophotometrically at 366 nm.

Apoptosis measurement for DNA damage was performed by Hoechst dye staining of fragmented nuclei. Cells grown on cover slips were treated with HG/HFA, fixed with 3.7% formaldehyde, and stained with Hoechst33342 (10 µg/mL) for 20 min at room temperature. The cover slips were then washed, mounted on glass slides, and analyzed by fluorescence microscopy. Cells with signs of apoptosis showed fragmented nuclei. DNA damage caused by ROS due to oxidative stress was also measured by DNA fragmentation using standard 2% agarose gel electrophoresis of damaged DNA, as described previously [25,29]. Apoptosis was also measured in HepG2 cells treated with HG/HPA by FACS analysis according to the vendor’s protocol, as described previously [25,26,27,28,29] Briefly, HepG2 cells treated with different doses of PA in the presence of normal/high glucose for 24 h were harvested, washed with PBS, and re-suspended (1 × 10^6^ cells/mL) in binding buffer (10 mM HEPES, pH 7.4, 140 mM NaCl, 2.5 mM CaCl_2_). A fraction (100 µL/1 × 10^5^ cells) of the cell suspension was incubated with 5 µL Annexin V conjugated to FITC and 5 µL propidium iodide (PI) for 15 min at 25 °C in the dark. A total of 400 µL of binding buffer was added to the suspension and apoptosis was measured immediately using a Becton Dickinson FACSCanto II analyzer. This method was able to distinguish the apoptotic cells from the viable and necrotic cells.

### 2.5. Measurement of GSH–Redox Metabolism

HepG2 cells were treated with different concentrations of palmitic acid in the presence of normal/high glucose for 24 h, as mentioned above. GSH/GSSG ratios and activities of GSH-metabolizing enzymes, GSH-Px, GSH-reductase, and glutathione S-transferase (GST) were measured in the HG/HFA treated and control cell extracts. GSH/GSSG ratios were measured using the GSH/GSSG-Glo kit as per the manufacturer’s instructions, as described previously [27,28]. GSH-Px activity using cumene hydroperoxide [30], GSH-reductase using oxidised glutathione (GSSG) [31], and GST activity using CDNB [32] as substrates were measured by standard protocols, as described previously [25,26,27,28].

### 2.6. Measurement of Catalase and SOD

Antioxidant enzymes, catalase, and SOD were measured using kits according to the vendor’s protocols. The measurement of catalase was based on its ability to catalyze the oxidation of methanol by hydrogen peroxide. The formaldehyde thus formed was measured colorimetrically in the presence of a chromogen at 450 nm.

The measurement of SOD was based on the conversion of xanthine to uric acid and hydrogen peroxide, in the presence of xanthine oxidase. The superoxide ions produced, in turn, reduced the NBT (nitroblue tetrazolium) to NBT-diformazan. The rate of the reduction with a superoxide anion is linearly related to the xanthine oxidase activity, and is inhibited by SOD. Thus, the percentage inhibition of SOD activity was measured colorimetrically at 550 nm.

### 2.7. Measurement of Cytochrome P450 Activities

Post-mitochondrial supernatants from control and HG/HFA-treated HepG2 cells were analyzed for CYP2E1, CYP3A4, CYP1A1, and CYP1A2 activities using N-nitosodimethylamine, erythromycin, 7-ethoxyresorufin, and methoxyresorufin, respectively, as substrates by standard protocols [33,34,35,36], as described previously [26,28].

### 2.8. Measurement of Mitochondrial Functions

Mitochondrial membrane potential was measured in cells after treatment with varying concentrations of glucose/palmitic acid for 24 h using a fluorescent cationic dye, DePsipher TM (R&D System Inc.), as described previously [25,26,27]. If the membrane potential is reduced, the dye cannot access the transmembranee space and remains in its green fluorescent form.

### 2.9. Measurement of Mitochondrial Bioenergetics

Freshly isolated mitochondria (5 µg protein) from untreated and HG/HFA-treated cells were suspended in 1.0 mL of 20 mM potassium phosphate buffer, pH 7.4, in the presence of the lauryl maltoside (0.2%). Oxidative phosphorylation enzymes, NADH-ubiquinone oxidoreductase (NADH-dehydrogenase, Complex I), and cytochrome c oxidase (Complex IV) were measured using the substrates coenzymeQ2 and reduced cytochrome c, respectively, according to the method of Birch-Machin and Turnbull [37], as described previously [25,26,27,28]. Krebs’ cycle enzymes, aconitase, and glutamate dehydrogenase were measured using the appropriate kits as per the vendor’s protocols, as described previously [27].

The ATP content in the HG/HFA-treated and untreated control cell lysates was determined using an ATP Bioluminescent cell assay kit according to the manufacturer’s recommended protocol, and samples were analyzed using the TD-20/20 Luminometer (Turner Designs, Sunnyvale, CA, USA).

### 2.10. Measurement of Hexokinase and Glucose-6-Phosphate Dehydrogenase (G6PDH) Activities

Activity of hexokinase, a cytosolic enzyme phosphorylating glucose to glucose 6-phosphate, was measured using the HK assay kit (Sigma-Aldrich, St. Louis, MO, USA) as per the vendor’s instructions. Additionally, activity of an NADPH-producing cytosolic enzyme, glucose 6-phosphate dehydrogenase (G6PDH), was measured in HepG2 cell extracts treated with HG/HPA using glucose-6-phosphate as the substrate by following the increase in absorption due to the reduction of NADP to NADPH at 340 nm.

### 2.11. Measurement of Inflammatory Markers, NO, and Lipid Accumulation

HepG2 cells were treated with HG/HFA, as described above, and inflammatory markers TNF-α and IL6 were measured in the media using ELISA kits from BD Pharmingen (BD Biosciences, San Jose, CA, USA) as described in the vendor’s protocols, as described previously [38].

For NO assay, cells (2 × 10^5^ cells/well) were cultured in six well plates for 24 h prior to HG/HPA treatments. NO production was determined by measuring the concentration of total nitrite in the culture supernatants using Griess reagent (R &D Systems Inc.).

To measure triglyceride lipid accumulation, Oil Red O staining was carried out by the method of Pittenger MF et al. [39] with sight modifications. Briefly, HepG2 cells were grown in six well plates at a density of 0.5 × 10^5^ cells/well and were treated with different doses of palmitic acid in the presence of normal or high glucose media for 24 h. The cells were washed with PBS and fixed with 3.7% formaldehyde for 30 min at room temperature. Fixed cells were then washed and treated with 60% isopropanol for 5 min. The cells were then stained with freshly prepared Oil Red O stain for 15 min. Excess stain was then washed thoroughly and stained with hematoxylin for nuclear staining for 1 min. Cells were again washed thoroughly and viewed using the EVOS XL Core Imaging system (40× magnification). Lipid droplets appear red and nuclei appear blue.

### 2.12. SDS-PAGE and Western Blot Analysis

Cell extract proteins (50 µg) were separated on 12% SDS-PAGE and electrophoretically transferred on to nitrocellulose paper by western blotting, as described previously [25,26,27,28]. The immunoreactive protein bands were visualized after interacting with primary antibodies against marker proteins. Beta actin was used as the loading control. Densitometric analysis was performed using the Typhoon FLA 9500 system (GE Healthcare, Uppsala, Sweden) and expressed as relative ratios normalized against actin or other proteins as appropriate.

### 2.13. Statistical Analysis

Results are expressed as mean + S.E.M. of three individual experiments. Statistical significance of the data was assessed using SPSS software (version 23) by analysis of variance followed by LSD post-hoc analysis. *p*-values < 0.05 were considered statistically significant.

## 3. Results

### 3.1. Effects of HG/HFA on Cell Survival, Oxidative Stress, and Apoptosis

**Figure 1:**
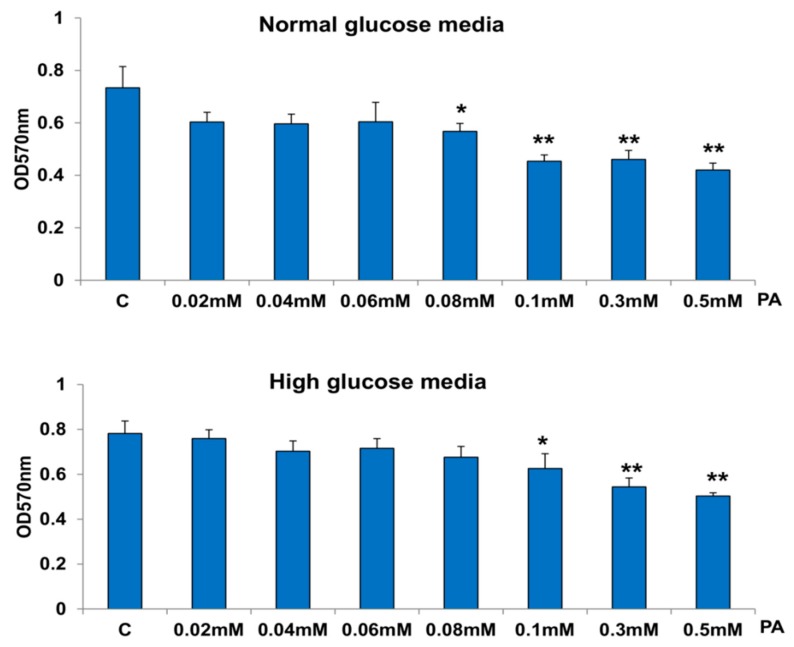
Effect of high glucose/high palmitic acid (HG/HPA) on cell viability. HepG2 cells (~2 × 10^4^) were treated with MTT as described in the Materials and Methods section, and viability was measured by formazan formation as a mitochondrial function of viable cells and measured at 570 nm. ‘C’ represents untreated control cells. The results are expressed as mean +/− SEM of three independent experiments. The asterisks indicate significant difference (* *p* ≤ 0.05, ** *p* ≤ 0.005,) from the control untreated cells.

Figure 1 shows a decrease in HepG2 cell viability with increasing concentrations of PA from 0.02 mM to 0.5 mM for 24 h, both in the presence of normal glucose as well as HG concentrations. The maximum inhibition (36–40%) was observed with the highest concentration of PA. No significant difference in the effects of PA was observed in normal glucose or HG treated cells. Based on our results and literature search, we selected 0.06 mM and 0.3 mM PA treatment for 24 h as the optimum doses for our further studies.

**Figure 2:**
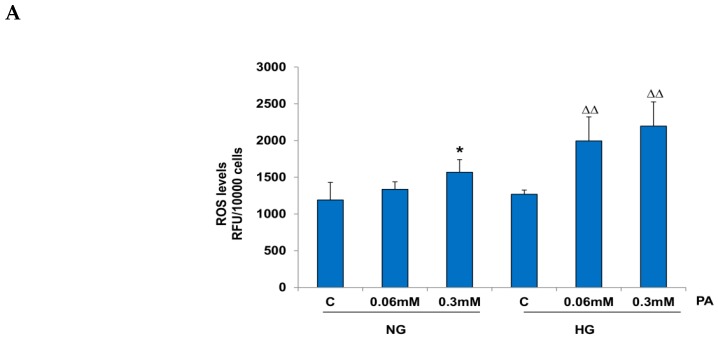

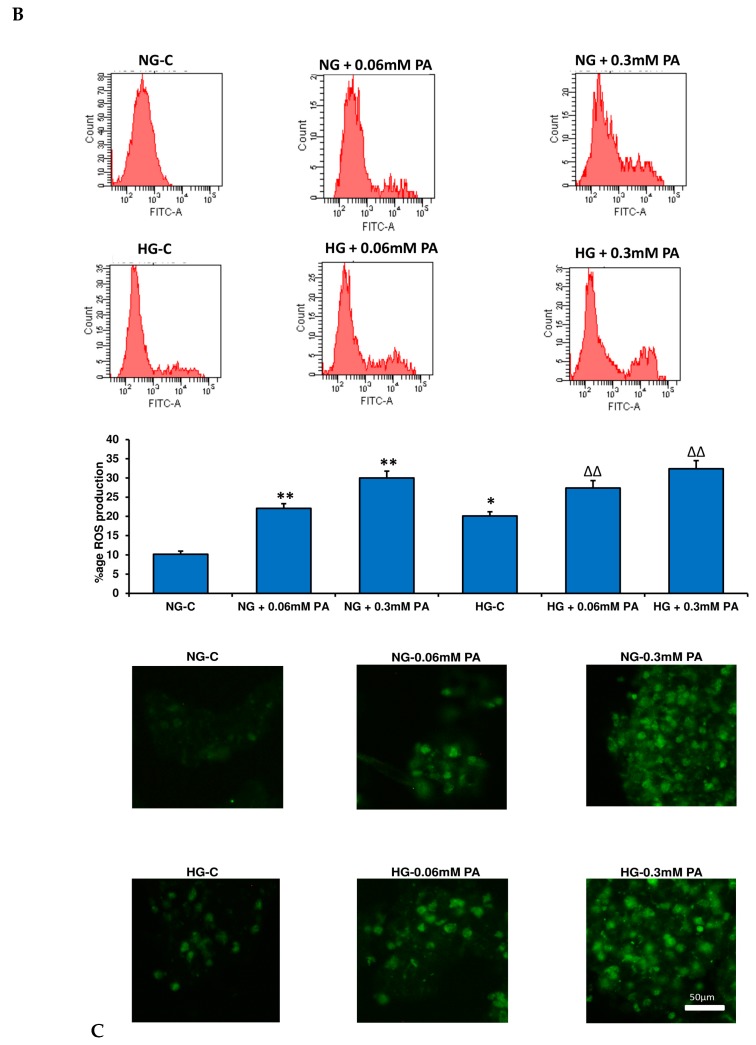
Effect of high glucose/high palmitic acid (HG/HPA) on reactive oxygen species (ROS) production. HepG2 cells were treated with high glucose/high palmitic acid, as described in the Materials and Methods section, and ROS were measured fluorimetrically (**A**) using the cell permeable probe 2′,7′-dichlorofluorescein diacetate (DCFDA), and by flow cytometry (**B**) using the FACSDiva software, as described previously. Production of ROS was also measured microscopically, in which cells grown on cover slips were incubated with DCFDA after high glucose/high palmitic acid treatment and fluorescence immediately visualized using an Olympus fluorescence microscope (**C**). Original magnification ×200. The results are expressed as mean +/− SEM of three independent experiments. Asterisks indicate significant differences (* *p* ≤ 0.05, ** *p* ≤ 0.005) relative to untreated control cells under normal glucose condition (NG-C), and triangles indicate significant differences (ΔΔ *p* ≤ 0.005) relative to untreated control cells under high glucose condition (HG-C).

A significant increase (31%) in ROS production was seen with 0.3 mM PA in the presence of NG, while 0.06 mM PA showed no appreciable effects. On the other hand, PA treatment markedly increased (60–70%) ROS production in the presence of HG (Figure 2A). These results suggest that PA treatment augments ROS production in the presence of high glucose. FACS and microscopic analysis (Figure 2B,C) confirmed the increased production of ROS with PA treatment.

**Figure 3:**
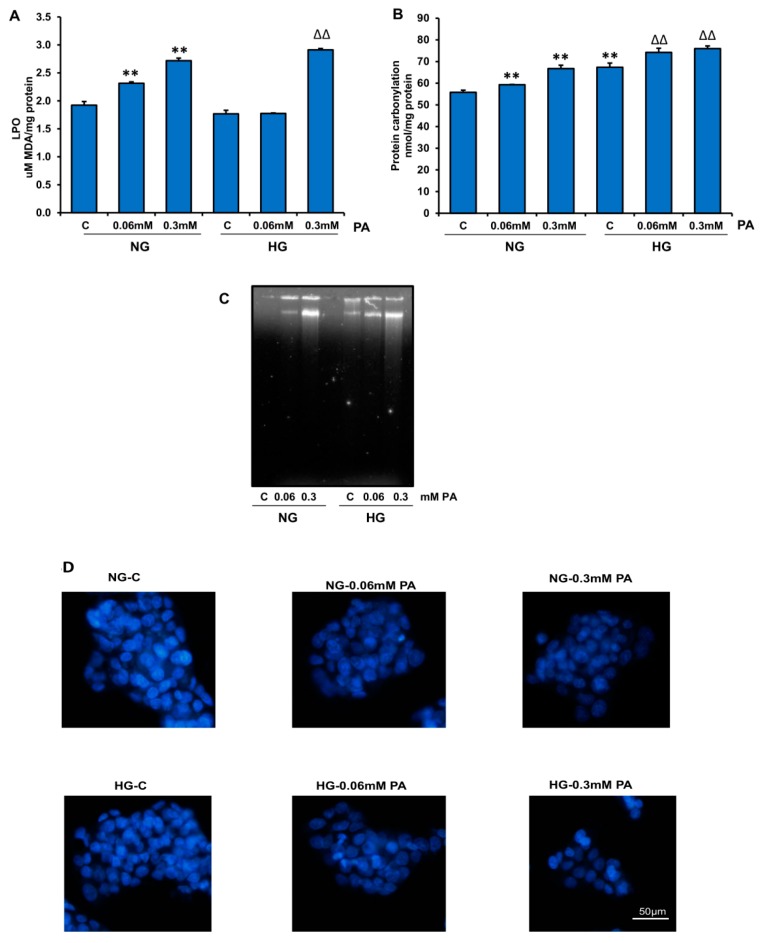
Effect of high glucose/high palmitic acid (HG/HPA) on lipids, proteins and DNA degradation. Lipid peroxidation (LPO) was measured as the total amount of malonedialdehyde (**A**), as per the vendor’s protocol (Oxis Research, Inc.). Protein carbonylation (**B**) was assayed by the dinitrophenylhydrazine (DNPH) method, as described in the Materials and Methods section. DNA fragmentation (**C**) was analyzed by agarose gel (2%) electrophoresis and ethidium bromide staining. DNA fragmentation was also analyzed microscopically (**D**) by staining the treated cells with Hoechst 33,342 dye, as described in the Materials and Methods section. Asterisks indicate significant differences (** *p* ≤ 0.005) relative to untreated control cells under normal glucose condition (NG-C), and triangles indicate significant differences (ΔΔ *p* ≤ 0.005) relative to untreated control cells under high glucose condition (HG-C).

Increased ROS production also resulted in the increased oxidative peroxidation of lipids (Figure 3A), proteins (Figure 3B), and DNA (Figure 3C), and this oxidative stress damage to lipids, proteins, and DNA by PA was more pronounced in the presence of HG.

In support of increased ROS production and oxidative-stress-related peroxidation, our results also demonstrated increased nuclear condensation (evident by decreased Hoechst nuclear staining Figure 3D) in cells treated with high PA in the presence of HG.

**Figure 4:**
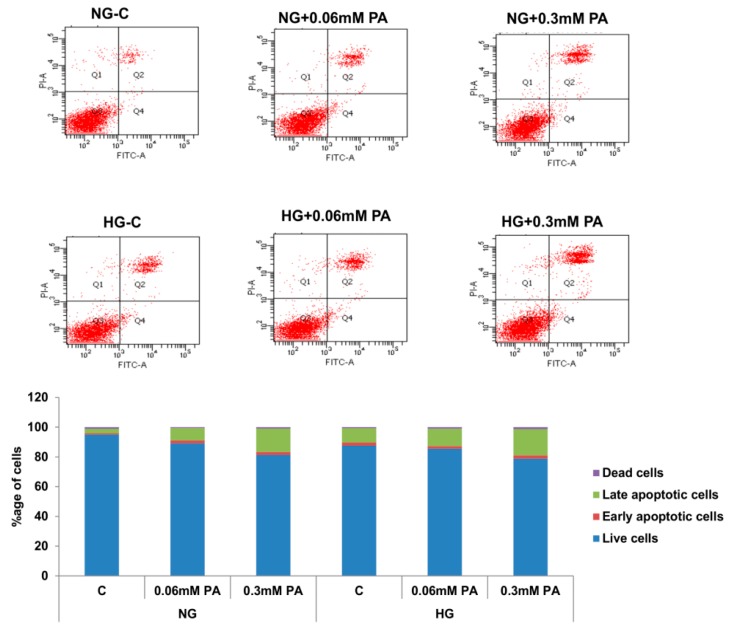
Effect of high glucose/high palmitic acid (HG/HPA) on apoptosis. Apoptosis was measured by FACS analysis, as described previously [25,26,27,28,29]. Histogram shows percentage of viable, early apoptotic, late apoptotic, and dead cells. Representative dot plots from three experiments are shown. The results are mean +/− SEM of three independent experiments.

A significant increase in the percentage of cells undergoing early/late apoptosis was also observed by FACS analysis (Figure 4).

### 3.2. Effects of HG/HFA on Antioxidant and Redox Metabolism

**Figure 5:**
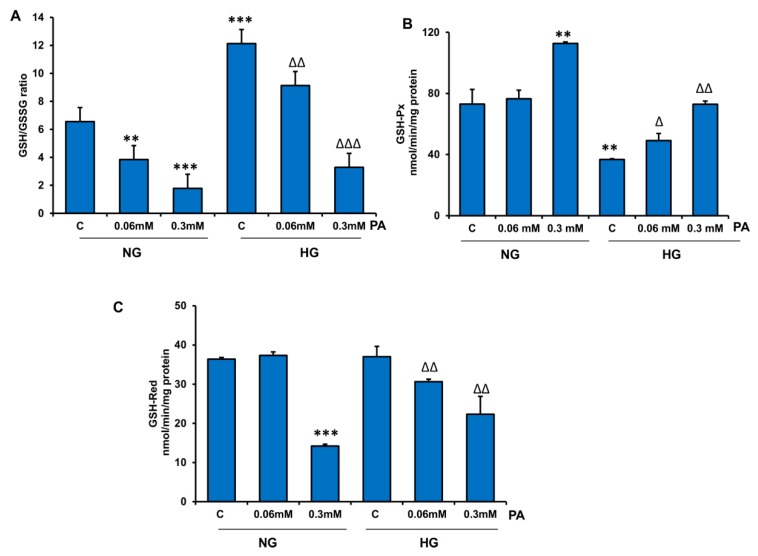
Effect of high glucose/high palmitic acid (HG/HPA) on glutathione (GSH) metabolism. HepG2 cells were treated with different doses of palmitic acid under normal and high glucose conditions. GSH/GSSG (reduced/oxidized glutathione) ratio (**A**), GSH-Px (glutathione peroxidase) (**B**), and GSH reductase (glutathione reductase) (**C**) were measured. Results are expressed as mean +/− SEM of three experiments. Asterisks indicate significant differences (** *p* ≤ 0.005, *** *p* ≤ 0.001) relative to untreated control cells under normal glucose condition (NG-C), triangles indicate significant differences (Δ *p* ≤ 0.05, ΔΔ *p* ≤ 0.005, ΔΔΔ *p* ≤ 0.001) relative to untreated control cells under high glucose condition (HG-C).

Figure 5A shows a marked reduction (40–70%) in the ratio of reduced (GSH) to oxidized (GSSG) glutathione with increasing concentrations of PA. Interestingly, GSH concentration was about two-fold higher in HepG2 cells treated with HG in comparison with NG-treated cells. PA treatment significantly reduced the ratio of GSH/GSSG, suggesting a reduction in antioxidant homeostasis by PA treatment. The activity of GSH-Px (Figure 5B) was significantly increased (50–90%) when cells were treated with 0.3 mM PA, under both normal and hyperglycemic conditions. This could be due to the increased level of peroxides. On the other hand, the activity of GSH-reductase, which catalyzes the reduction of oxidized glutathione, was significantly decreased (40–60%) in cells treated with 0.3 mM PA. A significant alteration was observed with 0.06 mM PA only in the presence of HG (Figure 5C).

**Figure 6:**
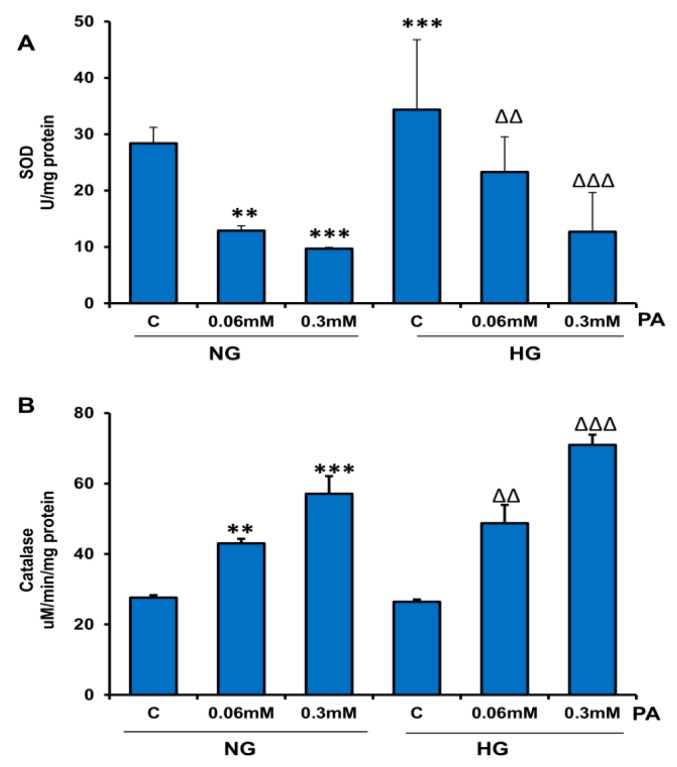
Effect of high glucose/high palmitic acid (HG/HPA) on the activities of antioxidant enzymes, superoxide dismutase (SOD), and catalase. HepG2 cells were treated with high glucose and high fatty acids, as described in the Materials and Methods section. SOD (**A**) was measured as percentage conversion of nitroblue tetrazolium (NBT) to NBT-diformazan according to the vendor’s protocol. Catalase measurement (**B**) was based on the formaldehyde produced, which was measured colorimetrically. Results are expressed as mean +/− SEM of three experiments. Asterisks indicate significant differences (** *p* ≤ 0.005, *** *p* ≤ 0.001) relative to untreated control cells under normal glucose condition (NG-C), and triangles indicate significant differences (ΔΔ *p* ≤ 0.005, ΔΔΔ *p* ≤ 0.001) relative to untreated control cells under high glucose condition (HG-C).

Similarly, activity of SOD, an antioxidant enzyme, was also significantly inhibited (30–60%) by PA treatments, particularly at higher concentrations of PA (Figure 6A). On the other hand, the activity of catalase, a hydrogen-peroxide-metabolizing enzyme, significantly increased with increasing concentration of PA (Figure 6B). These results suggest cellular adaptation in antioxidant and ROS metabolism in response to PA treatment in the presence of HG.

### 3.3. Effects of HG/HFA on CYP450s and Glutathione S-Transferase (GST) Enzymes

**Figure 7:**
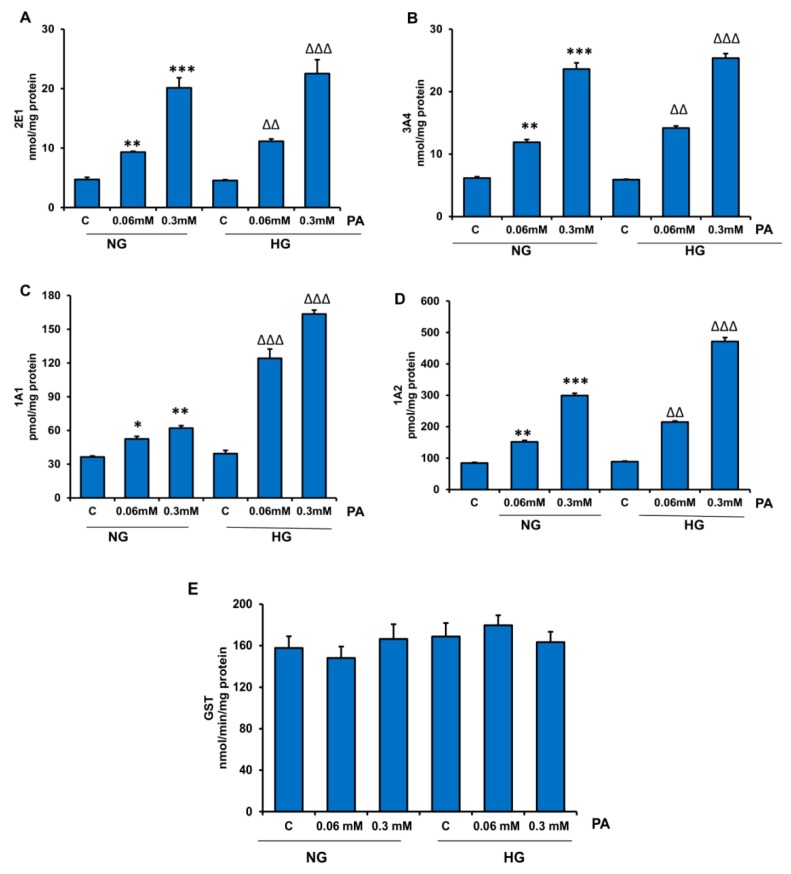
Effect of HG/HPA on CYP450 and GST activities. HepG2 cells were treated with high glucose and high fatty acids, and the activities of different CYP450 enzymes (**A**–**D**) and GST (**E**) were measured using the appropriate substrates, as described in the Materials and Methods section. Asterisks indicate significant differences (* *p* ≤ 0.05, ** *p* ≤ 0.005, *** *p* ≤ 0.001) relative to untreated control cells under normal glucose condition (NG-C), and triangles indicate significant differences (ΔΔ *p* ≤ 0.005, ΔΔΔ *p* ≤ 0.001) relative to untreated control cells under high glucose condition (HG-C).

CYP450s and GSTs play important roles in maintaining the redox homoeostasis and metabolite pools for many physiological substrates, especially lipids, and xenobiotics. Our results shown in Figure 7A–D demonstrate a marked increase (two–four-fold) in the activities of CYP2E1, CYP3A4, CYP1A1, and CYP1A2 by PA in NG and HG treated cells. GST activity, however, was not significantly altered after PA treatment (Figure 7E).

### 3.4. Effects of HG/HFA on Mitochondrial Functions

**Figure 8:**
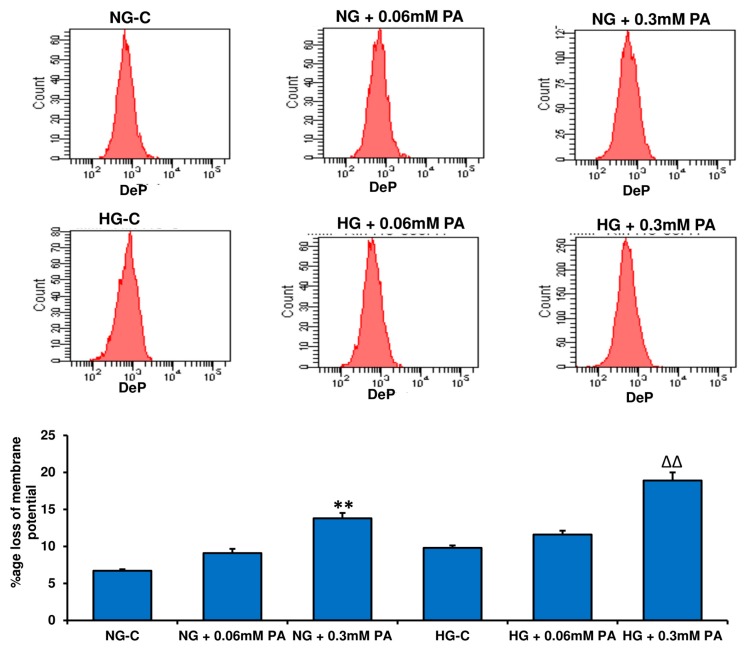
Effect of high glucose/high palmitic acid (HG/HPA) on mitochondrial membrane potential. HepG2 cells were treated with high glucose and high fatty acids, and mitochondrial membrane potential was measured by flow cytometry using a fluorescent cationic dye according to the vendor’s protocol (DePsipherTM, R &D System Inc.). A typical histogram representing the percentage loss of mitochondrial membrane potential is shown. Results are expressed as mean +/− SEM of three experiments. Asterisks indicate significant differences (** *p* ≤ 0.005) relative to untreated control cells under normal glucose condition (NG-C), and triangles indicate significant differences (ΔΔ *p* ≤ 0.005) relative to untreated control cells under high glucose condition (HG-C).

A significant loss of mitochondrial membrane potential was observed in HepG2 cells after PA treatment (Figure 8). HG treatment had a marginal effect on membrane potential disturbance.

**Figure 9:**
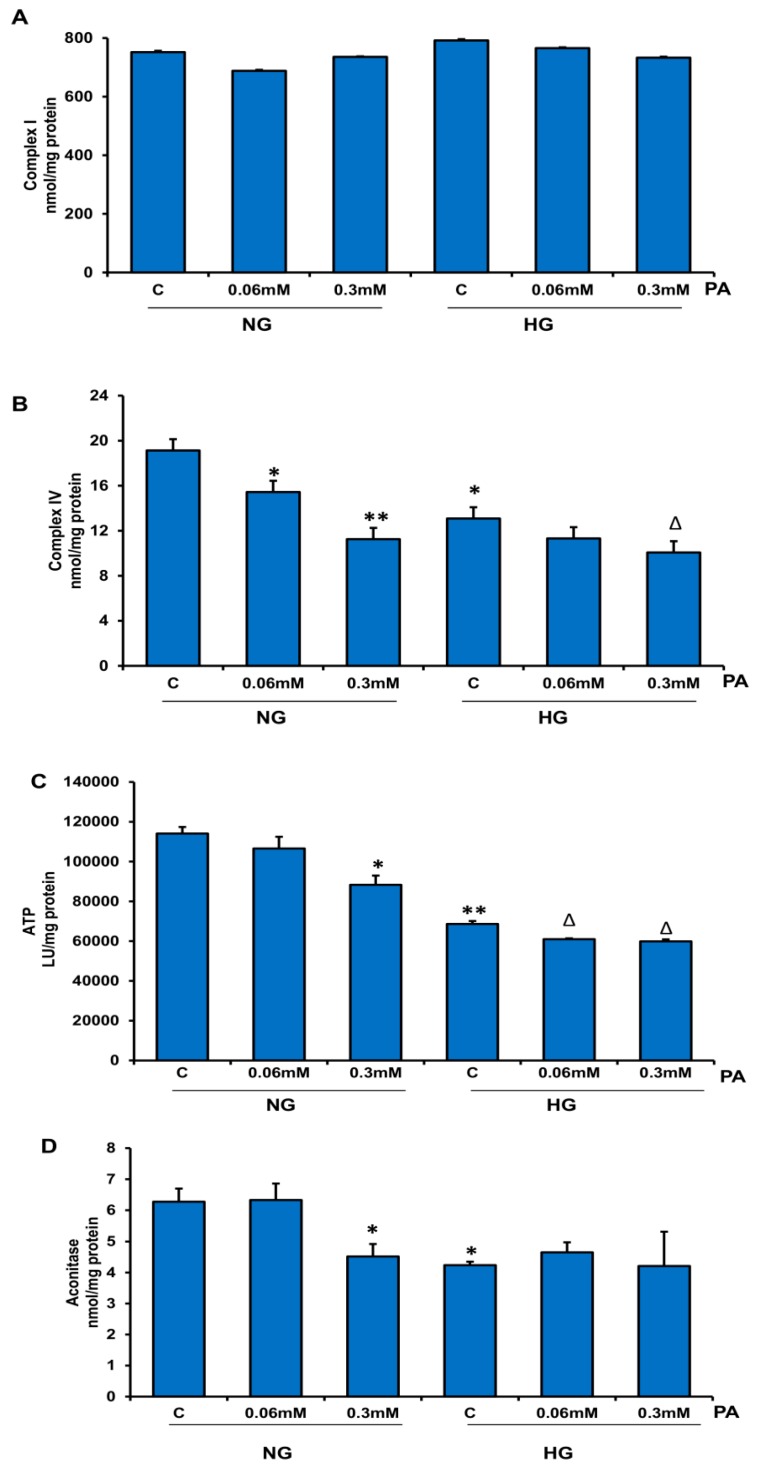
Effect of high glucose/high palmitic acid (HG/HPA) on mitochondrial enzymes and bioenergetics. HepG2 cells were treated with different doses of palmitic acid in the presence of normal and high glucose. Mitochondrial enzyme complexes, Complex I (**A**) and Complex IV (**B**), as well as ATP levels (**C**) and a ROS-sensitive enzyme, aconitase (**D**), were measured as described in the Materials and Methods section. Results are expressed as mean +/− SEM of three experiments. Asterisks indicate significant differences (* *p* ≤ 0.05, ** *p* ≤ 0.005) relative to untreated control cells under normal glucose condition (NG-C), and triangles indicate significant differences (Δ *p* ≤ 0.05) relative to untreated control cells under high glucose condition (HG-C).

No significant alterations were observed in the activity of NADH-dehydrogenase, Complex I (Figure 9A). However, the activity of respiratory Complex IV, cytochrome c oxidase, was inhibited (25–42%) by PA treatment in the presence of NG and HG (Figure 9B). HG treatment itself showed a marginal reduction in Complex IV activity. In agreement, ATP content was also significantly reduced after HG/PA treatment (Figure 9C). These results suggest that HG/PA inhibited mitochondrial respiration and oxidative phosphorylation of ADP, which could be a sign of metabolic reprograming by the cells to the high nutrient overload. A Krebs’ cycle enzyme, aconitase, which is disintegrated under oxidative stress conditions, also showed significant inhibition of its catalytic activity by 0.3 mM PA in the presence of NG. Significant inhibition was also observed with high glucose alone, which was not enhanced in the presence of PA (Figure 9D).

**Figure 10:**
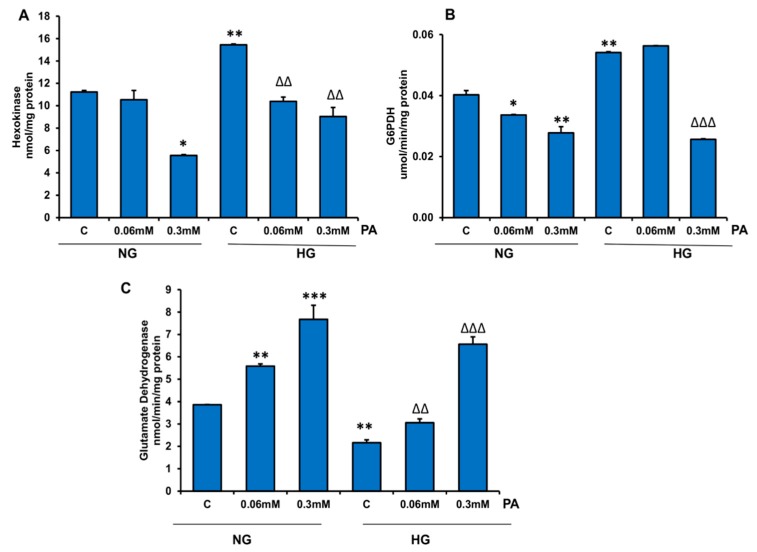
Effect of high glucose/high palmitic acid (HG/HPA) on cytosolic energy metabolizing enzymes. HepG2 cells were treated with high glucose and high palmitic acid, and the activities of hexokinase (**A**), glucose-6-phosphate dehydrogenase (G6PDH) (**B**), and glutamate dehydrogenase (GDH) (**C**) were measured as described in the Materials and Methods section. Results are expressed as mean +/− SEM of three experiments. Asterisks indicate significant differences (* *p* ≤ 0.05, ** *p* ≤ 0.005, *** *p* ≤ 0.001) relative to untreated control cells under normal glucose condition (NG-C), and triangles indicate significant differences (ΔΔ *p* ≤ 0.005, ΔΔΔ *p* ≤ 0.001) relative to untreated control cells under high glucose condition (HG-C).

In contrast, cytosolic metabolism of glucose by hexokinase and G6PDH seemed to increase when HepG2 cells were treated with HG alone (Figure 10A,B). However, treatment with PA at a higher concentration (0.3 mM) significantly inhibited the activities of these enzymes in the presence of NG and HG, suggesting adaptation and reprogramming in glycolytic glucose metabolism as well. Glutamate dehydrogenase (Glutamate to alpha-ketoglutarate direction) was significantly increased (Figure 10C) by PA treatment, suggesting the supply of metabolite for anaplerotic reactions in the Krebs’ cycle for metabolic adaptation of energy metabolism.

**Figure 11:**
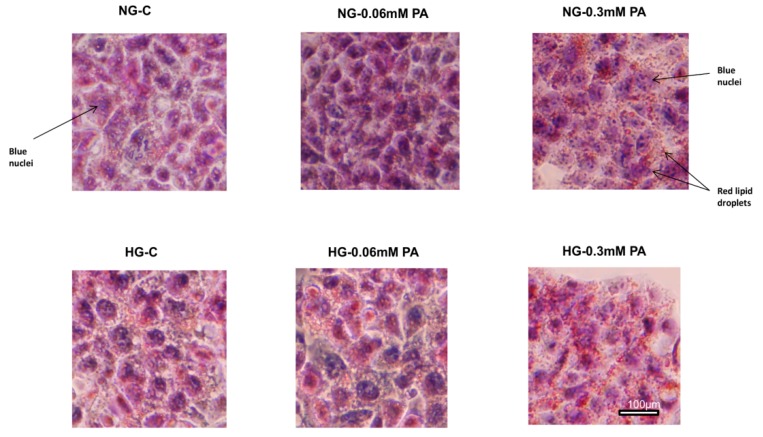
Effect of high glucose/high palmitic acid (HG/HPA) on lipid accumulation. HepG2 cells were grown on coverslips, treated with different doses of palmitic acid under normal and high glucose conditions, and stained with Oil Red O dye, as described in the Materials and Methods section. Hematoxylin was used for nuclear staining and viewed using the EVOS XL Core Imaging system (40× magnification). Lipid droplets appear red and nuclei appear blue.

HepG2 cells treated with HG/PA also exhibited increased deposits of TG-enriched lipid droplets in the cytosol (Figure 11) which again supports the metabolic reprograming towards anabolism as a measure to detoxify (store) nutrient overload and slow down the energy producing catabolic pathways.

**Figure 12:**
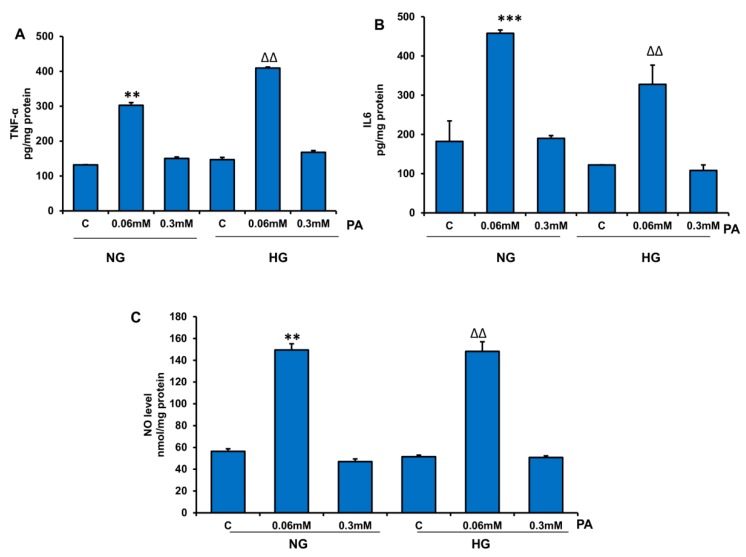
Effect of high glucose/high palmitic acid (HG/HPA) on inflammatory markers. HepG2 cells were treated with different doses of palmitic acid in normal/high glucose media, and inflammatory markers, TNF-α (**A**), IL6 (**B**), and NO levels (**C**) were measured in the media supernatants, as described in the Materials and Methods section. Results are expressed as mean +/− SEM of three experiments. Asterisks indicate significant differences (** *p* ≤ 0.005, *** *p* ≤ 0.001) relative to untreated control cells under normal glucose condition (NG-C), and triangles indicate significant differences (ΔΔ *p* ≤ 0.005) relative to untreated control cells under high glucose condition (HG-C).

HepG2 cells treated with 0.06 mM PA exhibited increased release of inflammatory markers TNF-α and IL6 in the presence of NG and HG (Figure 12A,B). Similarly, NO synthesis was also increased by PA treatment (Figure 12C). Surprisingly, not much alteration in inflammatory markers was observed with 0.3 mM PA.

**Figure 13:**
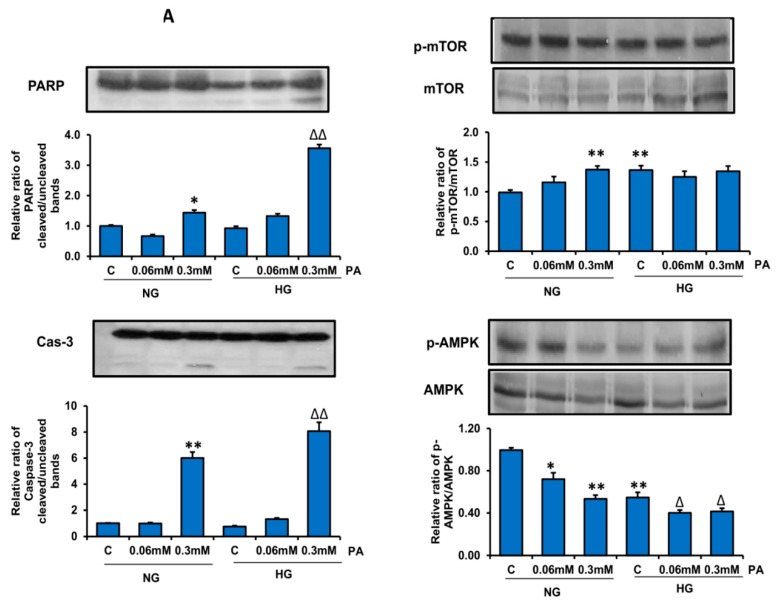

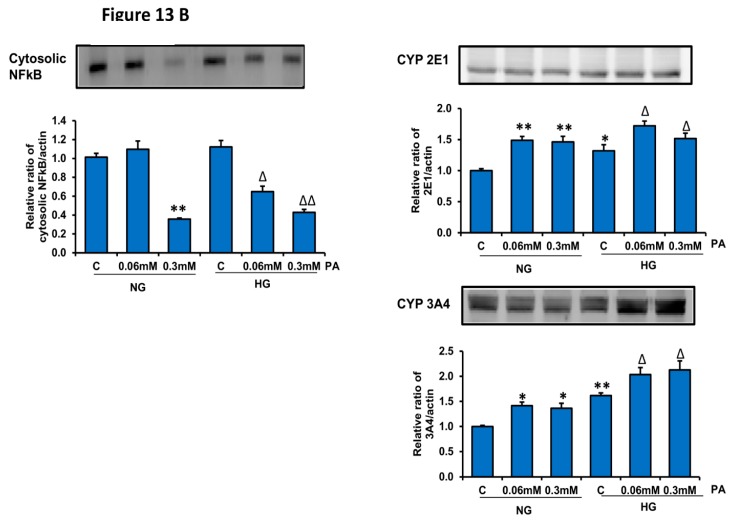
Effect of HG/HPA on protein expression. Samples of 50 ug protein from control and HG/HPA treated cellular extracts were separated on 12% SDS-PAGE gels and electroblotted onto nitrocellulose by western blot technique. The proteins were then detected using specific antibodies for PARP, caspase-3, mTOR, p-mTOR, AMPK, p-AMPK (**A**), and NFkB, CYP 2E1, and CYP 3A4 (**B**). Beta-actin was used as loading control. The quantitation of the protein bands is expressed as relative ratios normalized against actin or other specific proteins as appropriate. The figures are representative of three experiments. Asterisks indicate significant difference (* *p* ≤ 0.05, ** *p* ≤ 0.005) relative to untreated control cells under normal glucose condition (NG-C), (Δ *p* ≤ 0.05, ΔΔ *p* ≤ 0.005) relative to untreated control cells under high glucose condition (HG-C).

Figure 13A shows the expression of proteins involved in cell survival and energy sensing. As shown, increased cleavage of PARP and caspase-3 was observed, suggesting increased apoptosis when cells were treated with HG/HPA. Increased translocation of NF-kB from cytosol of HPA-treated cells also suggests an increase in inflammatory and redox signaling. A decrease in the phosphorylation of AMPK, a sensor of cell energy hemostasis and metabolism, with a concomitant increase in the phosphorylation of mTOR, an autophagy inhibitor, was observed under HG/HFA conditions. This could again be a metabolic adaptation of the cells to divert catabolism to anabolism for storage of glucose and lipids. In support of our results demonstrating the increased catalytic activities of CYP 2E1 (also a marker of increased oxidative stress in cells) and CYP 3A4 by HPA treatment, our results also demonstrated an increase in the expression of the corresponding proteins for CYP 2E1 and CYP 3A4 (Figure 13B).

## 4. Discussion

Glucolipotoxicity induces metabolic syndrome, as seen in insulin resistant type 2 diabetes, obesity, nonalcoholic fatty liver disease (NAFLD), and cardiovascular complications [12,40,41]. Both in vitro cell culture and in vivo animal studies have implicated oxidative stress, inflammation, and mitochondrial dysfunction caused by glucolipotoxicity in the progress of disease complications [8,13,42,43,44,45]. Our previous studies on hyperglycemia and hyperlipidemia models using STZ-induced type 1 and obese and non-obese animal models of type 2 diabetes have shown the involvement of oxidative stress and reduction in mitochondrial respiratory function and energy metabolism in multiple organ dysfunctions [45,46,47,48]. However, under in vivo conditions, the combined effects of glucolipotoxicity are difficult to evaluate, as it is a complex process and represents the effects of numerous physiological, endocrine, and environmental factors working in tandem, which cause alterations in energy metabolism and organ responses. Therefore, in analogy, we exposed HepG2 cells to high concentrations of glucose (25 mM) (glucotoxicity) and palmitic acid (0.3 mM), the most abundant saturated fatty acid (lipotoxicity) alone or in combination, and investigated inflammatory and oxidative stress responses in conjunction with metabolic changes in energy metabolism in these cells. Consistently with the numerous studies cited above, our results indicated increased production of ROS, oxidative damages to the lipids, proteins, and DNA, altered redox homeostasis, increased inflammatory responses, and adaptation of glycolytic and mitochondrial energy metabolism in response to HG and HPA, especially in combination. Our results also indicated that glucotoxicity and apoptosis were augmented in the presence of PA. Glucolipotoxicity also resulted in increased production of inflammatory markers IL6 and TNF-α and increased NO production under increased oxidative stress conditions, resulting in loss of mitochondrial membrane potential and respiratory complex IV activity. This, in turn, resulted in the reduction of ATP production and ROS-sensitive aconitase activity. These results have confirmed our previous finding using in vivo diabetic models of hyperlipidemia and hyperglycemia [45,46,47,48]. Glucolipotoxicity caused increased ROS production and DNA damage, which, in turn, augmented mitochondrial dysfunction and apoptosis in HepG2, as well as numerous other cell types [43,44,49,50,51,52]. In the present study, we also demonstrated that HPA caused inhibition of the glycolytic glucose metabolizing enzyme, hexokinase, and G-6-P-dehydrogenase activities, suggesting a reduction in glucose oxidative pathways. Previous studies [52] have also supported these findings, suggesting an adaptation in energy metabolism to reduce the catabolic production of ATP, and perhaps suggesting that the nutrient overload might be diverting the metabolism to anabolism (increased glycogen and triglyceride synthesis) as a way to detoxify (adapt to) HG/HPA toxicity. We also have demonstrated an increase in mitochondrial glutamate dehydrogenase (glutamate to alpha-ketoglutarate) and accumulation of lipid in HepG2 cells, indicating increased anabolic and anaplerotic pathways in nutrient overload, causing inhibition of autophagy and leading to increased apoptosis [13,15,16,21,53]. This observation was again supported by our results on HepG2 glucolipotoxicity, where we have shown an increased phosphorylation of mTOR and a simultaneous decreased phosphorylation of AMPK. Previous studies have also demonstrated a decrease in AMPK and increase in mTOR activities by glucolipotoxicity under increased inflammatory and oxidative stress (increased ROS) conditions due to nutrient overload, causing a decrease in autophagy and increased apoptosis with alterations in redox/ROS homeostasis [54,55,56]. This could again mean metabolic adaptation by the cells to divert catabolism toward anabolism for storage of glucose and triglyceride (as evidenced by the increased lipid accumulation). In our study on HepG2 cells, glucolipotoxicity by HG and HPA also induced NF-kB and other inflammatory markers, in addition to alterations in glutathione antioxidant metabolism and induction of the CYP450 detoxifying enzymes. Increased CYP3A4 and CYP2E1 activities have also been reported by us, as well as other researchers in hyperglycemia under diabetic conditions, as well as under in vitro conditions of nutrient overload [44,45,57].

## 5. Conclusions

Our results suggest that under nutrient overload conditions, in the presence of HG and high PA, a saturated fatty acid, HepG2 cells undergo severe metabolic and oxidative stress, causing increased ROS production, lipid, protein, and DNA damage, NF-kB-, TNF-α-, IL6-, and NO-dependent inflammatory responses, increased apoptosis, and decreased mitochondrial function leading to altered energy metabolism. The increased glucolipotoxicity also caused alterations in GSH-dependent redox metabolism, as well as in CYP-dependent metabolism of endogenous and exogenous compounds. NF-kB/AMPK/mTOR-dependent metabolic signaling appears, at least in part, to play a key role in the metabolic adaptation of cells under glucolipotoxicity conditions. These results might have implications in understanding and elucidating the molecular mechanisms of hyperglycemia/hyperlipidemia-induced complications in diabesity and cardiovascular diseases, as well as in identifying molecular targets for therapeutic management.

## Abbreviations

PA	palmitic acid
NG	normal glucose
HG	high glucose
HFA	high fatty acid
HPA	high palmitic acid
MTT	3-(4,5-dimethylthiazol-2-yl)-2,5-diphenyltetrazolium bromide
ROS	reactive oxygen species
CDNB	1-chloro 2,4-dinitrobenzene
DCFDA	2′,7′-dichlorofluorescein diacetate
LPO	lipid peroxidation
NO	nitric oxide
GSH	reduced glutathione
GSSG	oxidized glutathione
GST	glutathione S-transferase
GSH-Px	glutathione peroxidase
GSH-Red	glutathione reductase
SOD	superoxide dismutase
CYP	cytochrome P450
MMP	mitochondrial membrane potential
GDH	glutamate dehydrogenase
HK	hexokinase
G6PDH	glucose 6-phosphate dehydrogenase
TG	triglyceride
NF-kB	nuclear factor kappa-light-chain-enhancer of activated B cells
AMPK	AMP-activated protein kinase
mTOR	mammalian target of rapamycin
TNF-α	tumor necrosis factor alpha
IL6	interleukin 6
